# Unilateral Iris Transillumination Resembling BAIT Phenotype Following Contralateral Vitrectomy

**DOI:** 10.1155/crop/6612262

**Published:** 2025-03-05

**Authors:** Olivier Lambrechts, Luc Van Os

**Affiliations:** Ophthalmology, University Hospital Antwerp, Edegem, Belgium

**Keywords:** BAIT, iris transillumination, uveitis, vitrectomy

## Abstract

**Objective:** This study is aimed at describing a unilateral presentation of acute iris transillumination following contralateral vitrectomy with postoperative endophthalmitis.

**Methods:** This case study is based on the medical record of a patient who presented to our hospital in 2017.

**Results:** A 70-year-old female patient presented to our department with metamorphopsia and decreased vision in the right eye. She was diagnosed with a Stage 3 macular hole in the right eye for which she underwent 23G pars plana vitrectomy with gas tamponade. Postoperative topical treatment of tobramycin/dexamethasone was administered. Nine days after surgery, she presented to a different medical center with postoperative endophthalmitis in the right eye. Oral moxifloxacin was administered, an intravitreal injection with vancomycin was performed, and topical treatment with dexamethasone/chloramphenicol and neomycin/polymyxin B/dexamethasone was started. One month after surgery, she presented again to our department, this time with scleritis with associated anterior uveitis of the left eye. She was treated with oral ibuprofen, topical prednisolone acetate, and atropine sulfate, which resulted in clinical resolution. Three weeks after this episode, the left eye showed patchy transillumination of the iris matching the bilateral acute iris transillumination (BAIT) syndrome phenotype; however, the iris in the right eye remained normal.

**Conclusion:** To the best of our knowledge, this case is the first to show a unilateral phenotype of BAIT after contralateral vitrectomy. This suggests that previous vitrectomy, injection of vancomycin, or topical corticosteroids or chloramphenicol could be protective against the development of acute iris transillumination.

## 1. Introduction

Bilateral acute iris transillumination (BAIT) is a rare clinical presentation mostly seen in young to middle-aged women. Most reports on BAIT suggest a causative role of a preceding upper respiratory tract infection (including COVID-19) or previous use of moxifloxacin. The mechanism through which moxifloxacin might cause BAIT is still unclear, but a possible toxicity of moxifloxacin to iris melanocytes has been proposed [1]. Patients typically present with severe photophobia caused by the iris transillumination [2]. Other common complaints are eye pain, blurred vision, and conjunctival injection. Clinical examination may show ocular hypertonia, fixed semimydriatic pupils, and dispersion of pigment in the anterior chamber leading to deposits in the trabecular network. The clinical image may be falsely diagnosed as uveitis.

## 2. Case Presentation

A 70-year-old female patient presented to our department with metamorphopsia and decreased vision in the right eye. She was diagnosed with a Stage 3 macular hole in the right eye, and the left eye showed no abnormalities. She underwent 23G pars plana vitrectomy with peeling of the internal limiting membrane using infracyanine to dye the membrane. A gas tamponade with 20% SF_6_ was applied, and the patient was positioned with their head down postoperatively. A topical treatment of tobramycin/dexamethasone (Tobradex) six times daily was administered.

At 1 week postoperatively, anatomical success was achieved with the closure of the macular hole, but the patient continued to report metamorphopsia and had a BCVA of 0.12. There was a trace of cells in the anterior chamber and the presence of a gas bubble filling around 50% of the vitreous cavity. Otherwise, there were no abnormalities, and the left eye still appeared fully normal.

Nine days after surgery, the patient presented to the emergency department of a different hospital due to painless but progressive vision loss and the appearance of myriad floaters in the right eye for 1 day. A 4+ cell and a small hypopyon were present in the anterior chamber, and the vitreous appeared diffusely cellular on ultrasound. Intraocular pressure was 17 mmHg. A diagnosis of postoperative endophthalmitis was made, and an intravitreal injection with vancomycin was administered. Topical treatment was changed to chloramphenicol/dexamethasone (De Icol) hourly, neomycin/polymyxin B/dexamethasone ointment at night, and a 5-day course of moxifloxacin (Avelox) 400 mg orally was started. No vitreous biopsy was taken.

One month after the initial vitrectomy, she returned to our department complaining of severe pain in the left eye for 5 days, especially on lying down. The pain woke her up at night and increased on moving the eye. At clinical evaluation, there was marked redness with dilated and tortuous vessels superiorly on the left eye, with 2+ pigmented cells in the anterior chamber and a fixed semimydriatic pupil. No keratic precipitates were observed. The vision was unchanged with a BCVA of 1.0, and the intraocular pressure was 13 mmHg. We made a tentative diagnosis of scleritis with associated anterior uveitis and started treatment with oral ibuprofen 600 mg three times daily, topical prednisolone acetate eight times daily, and atropine sulfate three times daily. The treatment for the right eye was tapered to chloramphenicol/dexamethasone four times daily and neomycin/polymyxin B/dexamethasone ointment at night, as the inflammation in that eye had responded well to the previous treatment and was down to a trace of cells in the anterior chamber and a clear vitreous space. With treatment, the complaints and redness in the left eye promptly disappeared, and treatment could be tapered and stopped.

Blood analysis showed a normal blood count, kidney function, and ionogram. Protein electrophoresis was normal, and rheumatoid factor was negative, as were the anti-CCP antibodies. Antinuclear antibodies (ANAs) were marginally positive with a titre of 1:160. Other autoantibodies like ANCA, anti-ds-DNA, and antiphospholipid antibodies were all negative. In this case, a serological test was not performed.

**Figure 1:**
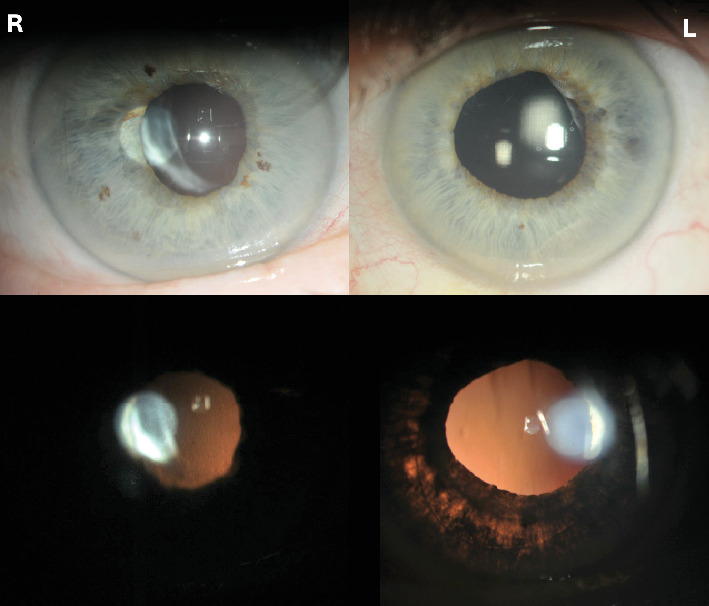
Pictures of the right and left eyes years after the initial presentation. The left eye clearly shows diffuse, patchy iris transillumination.

Three weeks after the inflammatory episode in the left eye, marked patchy iris transillumination became apparent in the left eye, where it had not been seen on any of the previous visits, despite being examined by several ophthalmologists. The iris transillumination is shown in Figure 1, and these pictures were taken on the patient's most recent examination.

## 3. Discussion

Our patient initially had an episode of presumed postoperative exogenous endophthalmitis in the right eye, 9 days after a vitrectomy. Unfortunately, no vitreous tap was performed, so there is no certainty about the infectious etiology of this inflammation. She was treated with intravitreal (vancomycin) and oral (moxifloxacin) antibiotics and intense topical steroids with a good response to this treatment. Two weeks after the inflammatory episode in the right eye, she developed scleritis with anterior chamber inflammation in the left eye, with no apparent underlying systemic disease. After the resolution of the scleritis, widespread iris transillumination and a fixed pupil became apparent in the left eye. The clinical image of the iris in the right eye remained completely normal. The clinical image for the left eye strongly resembles that of BAIT syndrome, apart from the fact that the presentation here is unilateral.

Only eight cases of unilateral presentation similar to BAIT are found in the literature. Most of them showed a unilateral BAIT-like syndrome after ipsilateral moxifloxacin injection with or without mitomycin application [3–6]. Besides intracameral moxifloxacin, systemic moxifloxacin may also cause unilateral iris transillumination, as seen in a study by Nascimento et al. [7]. The seventh case was seen after an ipsilateral respiratory sinus infection treated by oral azithromycin, although the etiology in this case is unclear, and a possibility of herpes simplex virus (HSV) is suggested [8]. The last case was seen during anterior uveitis supposedly caused by HSV [9]. A review of the literature did not reveal any reports of BAIT in vitrectomised eyes.

As the presentation of the iris transillumination in our case was unilateral but otherwise very similar to BAIT, it raises the question of whether there was a factor that protected the right eye from developing iris transillumination. The obvious differences between both eyes at the time of the inflammation in the left eye are that the right eye was vitrectomised, treated with local corticosteroids/antibiotics, and received a recent injection with vancomycin because of an exogenous endophthalmitis. In this hypothesis, the iris transillumination could have been secondary to the oral moxifloxacin treatment, as shown in the study by Nascimento et al. [7].

Another hypothesis could be the inflammatory episode in the left eye that was clinically diagnosed as scleritis with associated anterior uveitis. This inflammatory episode could also have been a viral anterior uveitis, although the iris transillumination in this case was very diffuse and not sectorial or localised as mostly can be seen in VZV or HSV anterior uveitis. However, the case by Nascimento et al. [7] showed presumed HSV-related uveitis with similar diffuse patchy iris transillumination. Furthermore, the clinical presentation was similar as the main complaints were pain and redness of the eye. We did not measure a raised intraocular pressure at any of the visits. Moreover, our patient did not suffer any previous episode of uveitis, so the diffuse iris transillumination would be very pronounced to be caused by just one episode of uveitis that promptly responded to treatment.

## 4. Conclusion

We report a case of unilateral acute iris transillumination matching the clinical image of BAIT, where the fellow eye recently presented a presumed exogenous endophthalmitis after vitrectomy but did not develop any iris transillumination.

This case might illustrate that there could be one or more protective factors preventing the fellow eye from developing the classic BAIT clinical image. Whether this was the previous vitrectomy, injection of vancomycin, or administration of topical steroids or chloramphenicol requires further investigation.

The patient provided oral informed consent that was documented in the presence of a witness.

## Data Availability

The data that support the findings of this study are available on request from the corresponding author. The data are not publicly available due to privacy or ethical restrictions.
